# Animal Welfare: Freedoms, Dominions and “A Life Worth Living”

**DOI:** 10.3390/ani6060035

**Published:** 2016-05-24

**Authors:** John Webster

**Affiliations:** Old Sock Cottage, Mudford Sock, Yeovil BA22 8EA, UK; john.webster@bris.ac.uk; Tel.: +44-117-928-9459

**Keywords:** animal welfare, quality of life, quality assurance

## Abstract

This opinion paper considers the relative validity and utility of three concepts: the Five Freedoms (FF), Five Domains (FD) and Quality of Life (QoL) as tools for the analysis of animal welfare. The aims of FF and FD are different but complementary. FD seeks to assess the impact of the physical and social environment on the mental (affective) state of a sentient animal, FF is an outcome-based approach to identify and evaluate the efficacy of specific actions necessary to promote well-being. Both have utility. The concept of QoL is presented mainly as a motivational framework. The FD approach provides an effective foundation for research and evidence-based conclusions as to the impact of the things we do on the mental state of the animals in our care. Moreover, it is one that can evolve with time. The FF are much simpler. They do not attempt to achieve an overall picture of mental state and welfare status, but the principles upon which they are based are timeless. Their aim is to be no more than a memorable set of signposts to right action. Since, so far as the animals are concerned, it is not what we think but what we do that counts, I suggest that they are likely to have a more general impact.

## 1. Introduction

The welfare of any sentient animal is determined by its individual perception of its own physical and emotional state [1]. This applies equally to the huge population of food animals as to the pets on whom we may lavish individual attention. Increasing public concern for action to improve animal welfare has generated the demand for animal welfare science that seeks to improve our understanding of the nature of animal emotions and motivation, and from this, improve the quality of our care. The animal welfare scientist has a responsibility not only to do research and publish papers to be read by other scientists, but also to communicate new knowledge and understanding in a manner that is most appropriate to the full spectrum of individuals in society; be they fellow scientists, those directly involved in the care of animals on farms, in laboratories, zoos and in the home, and finally those who may have little direct contact with animals but derive from them some utility or pleasure: *i.e.*, everybody else.

Animal welfare science is a big topic, since it embraces everything that may affect the physical and emotional state of the animal, its ability to cope and its quality of life. If we are to attempt a comprehensive analysis of the challenges to the welfare of a sentient animal and the consequences for its quality of life, we need some ground rules. David Mellor has, in this journal, recently summarised the thinking behind his development of the Five Domains [2] as a refinement of the concept of the Five Freedoms (FF) [3] and a framework for overall assessment of quality of life. The Five Domains (FD) are made up of four input categories: Nutrition, environment and health, categorised as survival-related factors, and behaviour, a situation-related factor that might better be described as opportunity to express rewarding behaviour, since behaviour itself is an outcome. The fifth domain is mental state, the outcome for the animal expressed in terms of negative and positive experiences and it is this domain that determines its welfare status.

The article by David Mellor [2] presents the FD as an alternative and a successor to the FF, developed “in the light of new scientific knowledge and understanding of animal welfare”. The editors of this journal have invited me, as the original proponent of the FF in their current form, to contribute an opinion piece to complement this article: in essence to discuss the relative validity and utility of the two approaches. My immediate, short answer is that it depends on who you are talking with: scientists, legislators, animal workers, or the general public. I recognise however that I need to explain this further.

## 2. The Five Freedoms: History

The phrase began life as the Four Freedoms, introduced by Franklin Roosevelt in his address to the US Congress in 1941. These he identified as freedom of speech, freedom of worship, freedom from want, freedom from fear. It should be obvious that these, like the later Five Freedoms, are aspirations. He was not making it an article of law that all the people should experience all of these perfect freedoms all of the time. They are, however, memorable.

The phrase was commandeered by the Brambell Committee report on the welfare of farm animals in intensive systems [4] to summarise their conclusion that farm animals in confinement should be allowed sufficient space to permit the following five minimal behaviours or activities, namely to stand up, lie down, turn round, stretch their limbs and groom all parts of the body. When in 1979 I joined the UK Farm Animal Advisory Committee (the predecessor of the Farm Animal Welfare Council, FAWC) I suggested that while these things were of vital importance to animals in the most intensive systems, they presented a very restricted view of farm animal welfare and left many, indeed most welfare problems off the page. Invited to come up with something more comprehensive I first proposed a new set of 5 Freedoms. FAWC worked on this original set and in 1993 published an updated version that matched each of the five freedoms with five provisions—and this is how they stand today [3].

- *Freedom from thirst, hunger and malnutrition*
  – By ready access to a diet to maintain full health and vigour
- *Freedom from thermal and physical discomfort*
  – By providing a suitable environment including shelter and a comfortable resting area
- *Freedom from pain, injury and disease*
  – By prevention or rapid diagnosis and treatment
- *Freedom from fear and distress*
  – By providing sufficient space, proper facilities and the company of the animal’s own kind
- *Freedom to express normal behaviour*
  – By ensuring conditions which avoid mental suffering

The alert will spot that these five freedoms are, in fact eleven. The key point is that they are all outcome measures. The provisions outline the husbandry necessary to promote these outcomes. The pan-European Welfare Quality^®^ assessment protocols for the welfare of farm animals [5] identify four welfare principles defined by twelve criteria. The FD recognise 15, or 18 “negative affects”, depending on how you count them [2]. Other publications have produced much longer lists; a process analogous to inflating the Ten Commandments into the Book of Leviticus, worthy but very dull. My case for the five freedoms has always been that, at a very simple and basic level, they are comprehensive. Moreover attempts to strengthen the case by adding detail can have the opposite effect since the more one tries to expand the argument by adding examples, the more likely one is to leave things out. This makes a very important point, especially in the context of legislation. The two key pieces of animal welfare legislation in the UK have an elegant simplicity [6]. The second definition of cruelty in the UK Protection of Animals Act 1911 states “to cause unnecessary suffering by doing, or omitting to do any act.” The recent UK Animal Welfare Act 2006 goes further by introducing a “duty of care” not only to avoid conditions that may lead to suffering but also to promote positive welfare. The strength of both these Acts, in my opinion, is that they stick to first, and timeless, principles.

## 3. The Five Freedoms: Limitations and Strengths

In his discussion of the five freedoms [2] Mellor acknowledges that they were not “intended to represent ideal or unattainable states… but to be a checklist by which to assess the strengths and weaknesses of husbandry systems” although he adds that “those who are less well informed (may consider) that such states of freedom are indeed fully achievable”. More seriously, he concludes that the concept of the five freedoms “does not capture, either in the specifics or the generality of its expression, the breadth and depth of current knowledge of the biological processes that are germane to understanding animal welfare and to guiding its management”. The five domains are presented as a concept that will capture all these things within its net.

Mellor [2] has proposed several limitations of the five freedoms. I have some more of my own. His strongest criticism is that, unlike the five dominions, they do not embrace the concept of positive welfare. I concede this point and shall return to it later. I also concede that as a practical series of recommendations for good husbandry, the five provisions, or the four welfare principles defined by Welfare Quality^®^ [5] may be of more direct practical use as a guide to humane husbandry than a simple statement of freedoms. However, I believe that the great strength of the FF in practice is that they describe outcome indicators. In recent years, outcome measures have become the standard approach to the development of quality control protocols, whether for the evaluation of animal welfare on individual farms or within overall production systems, e.g., the RSPCA Freedom Food scheme [7,8,9], or for the monitoring of human well-being in care homes [10]. In this sense, the FF concept was ahead of its time. I also concede that FF do not capture “the breadth and depth of current knowledge of the biological processes that are germane to understanding animal welfare and to guiding its management”—but then that was never their intention. I suggest we are getting into Leviticus territory here.

There are two ways in which, after 25 years reflection, I believe that the FF fall short. The first is that they only describe a snapshot: an attempt to define welfare at a moment in time. They do not properly reflect the causes and consequences of stresses that lead to long-term problems, e.g., behavioural problems such as learned helplessness due to long-term denial of normal behaviour in sows, or physical problems such as metabolic exhaustion in the dairy cow. The message here is that any outcome-based working protocol for the evaluation of animal welfare must include chronic indices of failure to cope with physical and emotional challenge.

My other concern relates to the fifth freedom “to express normal behaviour”. This is the only freedom ***to***, all others are freedoms ***from***, and, I believe, beyond cavil. Freedom ***to*** begs the question of what is normal behaviour? Does it include complete sexual freedom? Clearly no. Does it include freedom of one individual to compromise the welfare of another? I hope not. My freedom to swing my fist should stop at the point of your nose. The definition of normal behaviour can be interpreted quite sensibly so long as it doesn’t get too bogged down in sophistry. However in recent years I have come to believe that the fifth freedom would be more neatly expressed as “Freedom of Choice”. This incorporates freedom to express natural behaviour with regard to choice of diet, environment, social contact, comfort and security. Once again the concept of freedom of choice needs to be interpreted responsibly. Animals, like children in our care, should not, for example, be given free licence to eat themselves to death. I believe the concept of freedom of choice addresses all the concerns set out in the original Brambell report [4] into the welfare of farm animals in intensive systems (stand up, lie down, turn round, stretch their limbs and groom all parts of the body). More generally it addresses my greatest criticism of the business of factory farming, namely that by assuming more or less total control of the physical and social environment, we deny the animals the opportunity to make choices designed to promote their own quality of life.

## 4. The Five Domains: Strengths and Limitations

Briefly restated, the Five Domains approach identifies four categories of input factor that act upon the mind and body of a sentient animal then assesses their impact on a series of outcome indicators of mental state (15 negative and 13 positive affects). These may or may not then be integrated into an overall measure of welfare status. The individual outcome indicators provide a comprehensive structure upon which animal welfare scientists can build their knowledge and understanding of specific topics and identify topics for new research. They can also be used as the foundation for outcome-based protocols for the evaluation of animal welfare on farms, in zoos, research establishments *etc.*

Integration of the elements of the fifth domain into a single measure of welfare status may, in some circumstances, be necessary, e.g., for the overall classification of an individual farm as acceptable/unacceptable, or to give it a score within a ranking system as proposed by Welfare Quality^®^ [5] or the “5-Step Animal Welfare Standards” developed in North America [11]. For presentation to the general public any Quality Assurance scheme needs to provide an overall score as to acceptability or quality ranking, despite the difficulties inherent in offsetting a good score in one category against a poor score in another. However, whatever the overall score (unless everything is perfect), I argue that, so far as the animals are concerned, the most important purpose of any welfare-monitoring scheme is to identify and address specific problems. In this regard FF can be used to identify a comprehensive, specific, step by step, series of outcome indicators calling for action.

## 5. “A Life Worth Living”

The concept of Quality of Life (QoL), recognises that animals have both positive and negative experiences and focuses on the balance between the two. FAWC developed the notions of “a life not worth living”, “a life worth living” and “a good life [12]. Green and Mellor [13] formulated a four-tier QoL scaling system with two positive categories above and two negative categories below a neutral point of balance. Mellor [2] suggests that this approach is “more likely to be effective as a motivational framework than as an effective foundation for developing regulations”. I agree. I recognise the circumstances wherein an overall assessment of QoL can be of value, most obviously when a veterinarian is communicating with the owner of a pet faced by the prospect of euthanasia. I also recognise its utility as a basis for ranking farm overall animal welfare standards within quality assurance schemes [9,11].

I do suggest however that there are circumstances where the QoL concept is not particularly helpful and may indeed be counter-productive. My first concern is with the suggestion that it is possible to define QoL as the algebraic sum of positive and negative experiences. In the case of the dairy cow can one really quantify the extent to which (e.g.,) “affectionate sociability” can offset the pain of chronic lameness? More generally, we must acknowledge that our interpretation of the feelings of others can only be subjective. Since I can never be entirely sure how you are feeling, I am reluctant to speak with authority on the mental state of a dairy cow. In a strictly practical sense, one should, wherever possible, avoid the idea that a specific harm can be offset by another good. If there is a significant harm of any sort, efforts should be made to remedy it.

I also have my doubts about the concept of “a life worth living” because it is a value judgement made by us, rather than the animal in question. An insensitive farmer may consider that the life of a severely lame dairy cow has worth, so long as she continues to give milk. The highly sensitive owner of an infirm geriatric dog may consider that its life is worth prolonging because it continues to give and receive love. What these two extreme examples have in common is that in neither case does the animal contribute to the decision. The conclusion as to whether or not the life of a domestic farm or pet animal is worth living is something that we humans will make on behalf of the animal, based on how we think it feels when experiencing a physical and social environment largely dictated by us. A human teenager, exposed to such paternalism, or a family living under colonial rule may be fully justified in protesting “What right have you to tell me how I feel?” Thus, while I agree with Mellor that QoL may be effective as a motivational framework, so far as the animals are concerned, it is not what we think or feel but what we do that counts.

## 6. Five Freedoms *vs.* Five Domains: Who Needs Them?

My brief for this article was to discuss the relative validity and utility of FF and FD; the two approaches to the outline analysis of animal welfare. I hope by now I have made it clear that I believe the aims of FF and FD to be different but complementary. FD seeks to assess the impact (specific and overall) of the physical and social environment on the mental (affective) state of a sentient animal. FF (with the five provisions) is intended as an outcome-based approach to identify and evaluate the efficacy of specific actions necessary to promote well-being.

Both have utility. The Five Domains are clearly of use to animal behaviour and welfare scientists because they can embrace new knowledge and understanding, and provide pointers for new study. They can also be used for in-depth analysis of the impact of specific management practices (human actions) on animal welfare. For example, the FD approach has recently been used to evaluate the negative (adverse) welfare impacts of a range of procedures to which domestic horses may be subject, across a broad range of different contexts of equine care and training [14]. This has been a valuable exercise. In the case of procedures that may be deemed necessary, such as castration, it encourages us to think carefully as to what constitutes both best practice and minimally acceptable practice. For other procedures, such as the use of the whip in horse racing, it addresses the question as to whether the alleged “benefits” can ever justify the cost. In this and many other examples, the FD approach provides a highly effective foundation for research and evidence-based conclusions as to the impact of the things we do on the mental state of the animals in our care.

The Five Freedoms are much simpler (perhaps too simple for scientists) but are based on fundamental, timeless principles that do not need to be re-evaluated in the light of new research. They do not attempt to achieve an overall picture of mental state and welfare status. They are intended as no more than a memorable set of signposts to right action. Since, so far as the animals are concerned, it is not what we think or feel but what we do that counts, I suggest that they are likely to have more impact on, and be of more use to, everybody else—and that includes the animals.
